# Microbial reduction efficacy of various disinfection treatments on fresh-cut cabbage

**DOI:** 10.1002/fsn3.138

**Published:** 2014-06-30

**Authors:** Hyun-Hee Lee, Seok-In Hong, Dongman Kim

**Affiliations:** Korea Food Research InstituteGyonggi, Korea

**Keywords:** Disinfection, fresh-cut vegetables, microbiological safety, pathogen bacteria, sanitizer

## Abstract

To reduce the pathogenic microorganisms of fresh-cut vegetables, various sanitizers applied at different concentrations and against four different bacteria inoculated in high or low initial loads were tested on shredded cabbage. The bacteria were reduced by 1 log CFU g^−1^ after being exposed to over 100 ppm sodium hypochlorite, over 1% hydrogen peroxide, over 50 ppm peroxyacetic acid, and three types of electrolyzed water (EW) for 1 min. When the efficacy of the sanitizer was compared in the low initial bacterial load of 10^3^–10^4^ CFU g^−1^, a significant reduction in the inoculated bacteria was observed with the acidified EW treatment, followed by the 50 ppm peroxyacetic acid and the neutral EW treatment, which was more efficient than the 100 ppm sodium hypochlorite treatment. The efficacy of the various sanitizers used could be also influenced by different bacterial species.

## Introduction

Fresh-cut agricultural produce has a natural epiphytic microflora at harvest. The produce has the potential to be contaminated through processing, packaging, transportation, and retail from various sources, such as the environment or by humans. For this reason, fresh-cut produce is more likely to be influenced by spoilage and pathogenic contamination than whole produce (Doyle and Erickson 2008; Vandamm et al. 2013). However, the consumption of fresh-cut fruits and vegetables has rapidly increased with consumers' preference to prepare meals in a short time and to buy fresh produce to support a healthier lifestyle. In addition, this type of consumption can be affected by a national policy that promotes eating fresh vegetables in many countries (Abadias et al. 2008a,b). These individual and social changes in eating behaviors have increased the unexpected incidence of food-borne outbreaks caused by contaminated fresh fruit and vegetables in recent years. The pathogens most frequently associated with fresh vegetables include *Listeria monocytogenes*, *Salmonella* spp., *Shigella* spp., and enteropathogenic strains of *Escherichia coli*. These bacteria can exist on surfaces or even in the intercellular spaces of fresh produce in a mixed form (Abadias et al. 2008b; Doyle and Erickson 2008; Lynch et al. 2009).

To date, various disinfectants have been applied to whole or fresh-cut produce for sanitizing purposes (Artés et al. 2009; Ölmez and Kretzschmar 2009). Oxidative antimicrobials (e.g., chlorine, hydrogen peroxide, peracetic acid, etc.) are commonly used to reduce pathogenic bacteria and prevent cross-contamination by wash water. Chlorine is a very potent disinfectant with powerful oxidizing properties, and the use of chlorinated water at a decontamination stage in the washing of fresh-cut produce is widespread throughout the fresh produce industry because it is inexpensive and easy to perform. In the presence of organic matter in water, however, it may be deactivated and produce environmentally harmful by-products by reaction (Waters and Hung 2014). Hydrogen peroxide is widely used as a disinfectant in concentration ranges of 3–90% (v/v) and is considered environmentally friendly because water and oxygen are its sole reaction products. Peroxyacetic acid, a combination of acetic acid and hydrogen peroxide, is tolerant to several factors including a broad temperature range, pH (from 1 to 8), and soil contamination and is currently applied primarily in fruit and vegetable processing (Artés et al. 2009). With a strong bactericidal effect, electrolyzed water (EW) has been suggested as a valuable disinfection tool for the wash sanitation step in the fresh-cut industry (Huang et al. 2008). EW can be distinguished into several types, such as acidic oxidizing water (AcEW), alkaline reducing water (AlEW), and weak-alkalized or neutral water (NEW), that have different hypochlorite concentrations, pH, and oxidation–reduction potentials (ORP). Particularly due to its neutral pH, NEW does not contribute as aggressively as AcEW to the discoloration of fresh produce and is also more stable because loss of effective chlorine is significantly reduced at a pH of 6–9 (Abadias et al. 2008b).

Sanitizer's effectiveness on bacterial reduction is different depending on the product type, target microorganisms, time interval between contamination and washing, and treatment time (Gil et al. 2009). In addition, an effective sanitizer treatment may have a detrimental influence on the appearance of fresh produce. In this aspect, it is necessary to evaluate the microbial reduction efficacy of several disinfectants typically used in fresh-cut industry and the susceptibility of various food-borne bacteria, which can be cross-contaminated on fresh produce, under the same testing conditions. In this study, various sanitizing solutions were applied to shredded cabbage, which was inoculated with four pathogenic bacteria, for the purpose of investigating the effectiveness of the decontaminating treatments on the microorganisms of such a commodity.

## Materials and Methods

### Preparation of cabbage samples

Cabbages (*Brassica Oleracea* L. var. *capitata*) cultivated in Jeju Island during the winter and Gangwon province in the summer (both in South Korea) were purchased from a wholesale market in Seoul and held at 4°C and 97 ± 3% relative humidity (RH) until they were used. The cabbages were trimmed of the two outermost leaves and their cores and were shredded into strips of 10 × 120 mm using a sharp knife. Samples of about 50 g of the cabbage subjected to inoculation were placed into aseptic sampling bags (B01195WA; Nasco, Fort Atkinson, WI).

### Microorganisms and inoculation

Each bacterial strain was obtained from the microbial culture collection at the Korea Food Research Institute (Table 1). Authorized selective media were used for the isolation and cultivation. At first, four strains were cultured in the tryptic soy broth (Difco, Detroit, MI) at 37°C for 24 h, respectively, for making mother culture. Each mother culture was transferred individually into an appropriate broth and cultured at 37°C for *S. aureus* and *L. monocytogenes*, and at 30°C for *E. coli* O157:H7 and *S*. Typhimurium until the late log phase. Four pathogenic cultures were mixed together at the same proportion to make the inoculation cocktails (∼10^8^ or 10^6^ CFU mL^−1^). According to the method of Koseki et al. (2003), a given amount (0.5 mL) of the cell cocktails was sprinkled on the shredded cabbage (50 g) in a sampling bag on a clean bench. Then, the bag contents were gently shaken for 1 min to ensure an even distribution of the bacteria on the produce. The inoculated cabbage was stored at 5°C (85–90% RH) for 16–18 h to immobilize the organisms uniformly with a final inoculation level of 10^3^–10^4^ or 10^5^–10^6^ CFU g^−1^. The concentration applied was confirmed by plating 0.1 mL of the appropriately inoculated cabbage on each selective agar plate. The initial total microbial loads in the shredded cabbage before inoculation were less than 10^3^ CFU g^−1^.

**Table 1:** Test microorganisms and their selective media used.

Test microorganisms	Selective media
*Escherichia coli* O157:H7 (ATCC-43895)	Sorbitol MacConkey agar (Difco, Detroit, MI)
*Salmonella* Typhimurium (ATCC-14028)	XLT4 agar with 0.46 mL/100 mL supplement (Merck, Darmstadt, Germany)
*Staphylococcus aureus* (ATCC-14458)	Baird–Parker medium with 5 mL/100 mL egg yolk tellurite emulsion (Oxoid, Cambridge, UK)
*Listeria monocytogenes* (ATCC-19111)	Oxford Listeria selective agar with 1 mL/100 mL supplement (Merck)

### Preparation of disinfectant solutions and dipping treatment

Various disinfectant solutions were prepared as follows: 100, 200, and 450 ppm sodium hypochlorite (4.5% active chlorine content, Clean Rox, Yuhan Clorox Co., Inchon, Korea); 0.25%, 0.5%, 1%, and 2% (v/v) hydrogen peroxide (30 wt.% solution in H_2_O_2_, Sigma Aldrich, St. Louis, MO); and 50, 100, and 150 ppm peroxyacetic acid (32 wt.% solution in dilute acetic acid, Sigma Aldrich) were prepared by diluting the solutions in deionized water. Three kinds of fresh EW (AcEW, AlEW, and NEW) were generated using a flow-type electrolysis equipment (Acera 2000; Suchang Tech Co., Seongnam, Korea). Following manufacturer's instructions, a saturated sodium chloride solution was pumped into the generator at 0–10 mL min^−1^ and the current passing through the EW generator was set at 20–23 A. The pH and ORP of the tested solutions were measured with a pH meter (AR15; Fisher Scientific, Leicester, UK) and an ORP meter (RE-12P; TOA Electronics Ltd., Tokyo, Japan), respectively. The initial concentration of the available chlorine in the tap water, sodium hypochlorite, and EW used in this study was measured by the standard method of KFDA (2013). The inoculated shredded cabbage, in 50 g amounts, was soaked in 500 mL of each treatment solution for 1 min. After treatment, it was rinsed with tap water for 30 sec, allowed to drain on the clean bench for 5 min, and packaged into sterile bags for the experiment. A cabbage sample of 50 g was also soaked into 500 mL of tap water for 1 min as a control.

### Microbial analysis

The cabbage samples were mixed with 100 mL of 0.85% sterile NaCl solution and then homogenized with a stomacher (Bagmixer®400; Interscience, Bretèche, France) for 1 min. After homogenization, 1 mL of samples was serially diluted in 9 mL of 0.1% sterile peptone water and aliquots (0.1 mL) of the samples or diluents were surface plated onto each selective agar. All the agar media were incubated at 37°C for 24–48 h as appropriate, and then the viable cell colonies were counted and represented as log colony forming units (CFU) per gram of samples. Colonies randomly selected from the colonies with typical characteristics of the inoculated bacteria on selective media were confirmed biochemically with API strips (API Staph for *S. aureus*, API Listeria for *L. monocytogenes*, and API 20E for *E. coli* O157:H7 and *S*. Typhimurium, BioMérieux, Marcy l'Etoile, France) for the respective inoculated bacteria. The microbial reduction was expressed as the log difference in the viable cell counts before and after treatment.

### Statistical analysis

All of the experiments were carried out independently in triplicate and three analyses per replication at least were done. The significant differences in the experimental data among the disinfection treatments were analyzed using the ANOVA procedure (SAS Institute Inc., Cary, NC) at *P* < 0.01 with mean separation determined by Tukey's multiple range tests.

## Results and Discussion

### Effect of disinfectant type on the microorganisms on shredded cabbage

Table 2 shows the results of viability and variation in various bacterial strains for all treatments. In this study, dipping the samples into tap water for 1 min resulted in mean population reductions of less than 0.3 log CFU g^−1^ for all of the tested strains. All chemical treatments significantly lowered the various pathogens compared to the untreated and tap water sample. The sodium hypochlorite solution at 100–450 ppm significantly reduced the total population of the tested bacteria, resulting in reductions of 0.6–1.82 log CFU g^−1^. Among the various inoculated bacteria, the reduction in *S*. Typhimurium by ∼1.45–1.82 log CFU g^−1^ was higher than that of other bacteria by ∼0.6–1.50 log CFU g^−1^. However, there was a yellowish discoloration on the cabbage surface after dipping at over 200 ppm (data not shown).

**Table 2:** Viable cell counts and microbial reduction in the shredded cabbage treated with various disinfectant solutions at different concentrations (log CFU g^−1^).

Disinfectant type	Treatment	*Escherichia coli* O157:H7	*Salmonella* Typhimurium	*Staphylococcus aureus*	*Listeria monocytogenes*
NaOCl	Before treatment	5.69 ± 0.27a	5.70 ± 0.38a	5.70 ± 0.04a	5.53 ± 0.43a
	Tap water	5.44 ± 0.21ab (0.25)	5.41 ± 0.55a (0.29)	5.42 ± 0.05b (0.28)	5.30 ± 0.35a (0.23)
	100 ppm	5.09 ± 0.17bc (0.60)	4.25 ± 0.48b (1.45)	4.80 ± 0.07c (0.90)	4.45 ± 0.34b (1.08)
	200 ppm	4.81 ± 0.20cd (0.88)	4.09 ± 0.49b (1.61)	4.68 ± 0.06d (1.02)	4.24 ± 0.38b (1.29)
	450 ppm	4.63 ± 0.30d (1.06)	3.88 ± 0.31b (1.82)	4.20 ± 0.08e (1.50)	4.09 ± 0.41b (1.44)
HP	Before treatment	5.90 ± 0.20a	5.58 ± 0.71a	6.22 ± 0.18a	6.30 ± 0.12a
	Tap water	5.68 ± 0.12ab (0.22)	5.02 ± 0.31ab (0.56)	6.07 ± 0.16ab (0.15)	6.16 ± 0.09ab (0.15)
	0.25%	5.57 ± 0.09bc (0.33)	4.99 ± 0.10ab (0.59)	5.90 ± 0.16bc (0.32)	6.06 ± 0.14bc (0.24)
	0.5%	5.36 ± 0.15cd (0.54)	4.81 ± 0.18ab (0.77)	5.73 ± 0.12cd (0.50)	5.97 ± 0.13bc (0.34)
	1%	5.34 ± 0.08d (0.56)	4.76 ± 0.21ab (0.82)	5.75 ± 0.20cd (0.47)	5.88 ± 0.12cd (0.42)
	2%	5.03 ± 0.03e (0.87)	4.62 ± 0.19b (0.96)	5.48 ± 0.07d (0.75)	5.71 ± 0.15d (0.59)
PAA	Before treatment	5.10 ± 0.04a	5.10 ± 0.56a	5.46 ± 0.01a	5.33 ± 0.24a
	Tap water	4.92 ± 0.07b (0.18)	4.87 ± 0.47a (0.23)	5.23 ± 0.01b (0.23)	5.27 ± 0.17a (0.06)
	50 ppm	4.56 ± 0.09c (0.54)	4.01 ± 0.34b (1.09)	4.41 ± 0.02c (1.04)	4.50 ± 0.09b (0.83)
	100 ppm	4.26 ± 0.03d (0.84)	3.72 ± 0.25b (1.38)	4.27 ± 0.04d (1.19)	4.35 ± 0.23bc (0.98)
	150 ppm	4.21 ± 0.03d (0.89)	3.56 ± 0.24b (1.54)	4.17 ± 0.04e (1.28)	4.16 ± 0.15c (1.17)
EW	Before treatment	5.38 ± 0.34a	5.50 ± 0.20a	6.08 ± 0.55a	6.08 ± 0.37a
	Tap water	5.06 ± 0.29a (0.32)	4.52 ± 0.20b (0.98)	5.84 ± 0.52ab (0.24)	5.92 ± 0.37a (0.16)
	NEW	4.53 ± 0.36b (0.85)	3.82 ± 0.20c (1.68)	5.09 ± 0.54c (0.99)	5.30 ± 0.49b (0.78)
	AlEW	4.52 ± 0.32b (0.86)	3.72 ± 0.20c (1.78)	5.40 ± 0.65bc (0.68)	5.33 ± 0.41b (0.75)
	AcEW	4.47 ± 0.32b (0.91)	3.58 ± 0.20c (1.92)	4.99 ± 0.62c (1.09)	5.04 ± 0.43b (1.04)

Each value is the mean of three replicates with the standard deviation in three independent experiments. Any two means in the same column followed by the same letter are not significantly (*P* > 0.01) different by Tukey's multiple range tests. The log difference in the viable cell counts after treatment compared to the initial inoculation is expressed as the number in the parenthesis. NaOCl, sodium hypochlorite; HP, hydrogen peroxide; PAA, peroxyacetic acid; EW, electrolyzed water; AcEW, acidified electrolyzed water (pH 2.71 ± 0.19, 1,152 ± 20 ORP, 111.6 ± 15.7 ppm of free chlorine); NEW, neutral electrolyzed water (pH 8.43 ± 0.02, 670 ± 23 ORP, 99.6 ± 7.4 ppm of free chlorine); AlEW, alkaline electrolyzed water (pH 10.43 ± 0.42, 211 ± 13 ORP, 52.9 ± 13.8 ppm of free chlorine).

When hydrogen peroxide solutions of 0.25–2% (v/v) were applied to the cut cabbage, the efficacy of <1 log reduction was obtained along with surface browning at the high concentration (2%). Among the inoculated bacteria, the reduction in *S*. Typhimurium by 0.59–0.96 log CFU g^−1^ was relatively high and that of *L. monocytogenes* by 0.24–0.59 log CFU g^−1^ was comparatively low. For the peroxyacetic acid treatment, the bacterial population decreased by ∼1 log CFU g^−1^ at 50–100 ppm for 1 min, which coincides with the previous observation (Artés et al. 2009). Additionally, this reduction was similar to that obtained when the shredded cabbage was dipped into 100 ppm of the sodium hypochlorite solution. Among the bacteria tested, *S*. Typhimurium and *S. aureus* were more sensitive to the treatment, whereas *E. coli* O157:H7 was less sensitive. A severe discoloration occurred on the surface of shredded cabbage after dipping at over 100 ppm peroxyacetic acid (data not shown). Increasing concentration of various disinfectant solutions resulted in a greater population reduction, but the microbial reduction did not show linearity in proportion to the concentration (Delaquis et al. 2004).

In the case of EW treatments, the AcEW showed a log reduction range of 0.91–1.92 and had a relatively higher microbial reduction than the others. Depending on the inoculated bacteria, the NEW and AlEW exhibited 0.78–1.68 log and 0.68–1.78 log reductions, respectively. The microbial reduction obtained was greater for *S*. Typhimurium (ca. 1.68–1.92 log CFU g^−1^), whereas there was no significant difference among other inoculated bacteria (ca. 0.68–1.09 log CFU g^−1^). Hypochlorous acid (HOCl) is the primary active agent of EW and is the form of free available chlorine that has the highest antimicrobial activity against a broad range of microorganisms. The antibacterial ability of the AcEW can be explained by the high free chlorine concentration, high ORP, and low pH (Huang et al. 2008).

For the above treatments, the appropriate concentrations that showed the efficacy of a microbial reduction of ∼1 log CFU g^−1^ without severe visual deterioration or discoloration in the cabbage were selected and applied to the lower initial microbial load experiments of 10^3^–10^4^ CFU g^−1^. The selected sanitizing treatments included 100 ppm sodium hypochlorite, acidified, neutral, and alkaline EW (AcEW, NEW, and AlEW), 50 ppm peracetic acid, and 1% hydrogen peroxide.

### Effect of the initial microbial load on the disinfection of shredded cabbage

For the low initial load, the greatest reduction in inoculated bacteria was observed with AcEW by 0.79–1.41 log CFU g^−1^, followed by 50 ppm peroxyacetic acid with 0.97–1.18 log CFU g^−1^ and NEW with 0.69–1.34 log CFU g^−1^ reduction (Table 3). These treatments were more effective than 100 ppm sodium hypochlorite, with microbial reductions ranging from 0.70 to 1.13 log CFU g^−1^. The smallest reduction was obtained after a 1% (v/v) hydrogen peroxide treatment. AlEW, with microbial reductions ranging from 0.59 to 1.04 log CFU g^−1^, showed a slightly higher reduction than hydrogen peroxide treatment and a similar reduction to the 100 ppm sodium hypochlorite solution. Among the bacteria, *S*. Typhimurium and *E. coli* O157:H7 were more sensitive to the various dipping treatments than the others with a similar reduction. The sanitizers used in this study could not completely eliminate the inoculated bacteria of the low initial load experimental groups as reported previously (Koseki et al. 2003).

**Table 3:** Viable cell counts and microbial reduction in the shredded cabbage with high and low inoculation levels treated with different disinfectant solutions (log CFU g^−1^).

Treatment	*Escherichia coli* O157:H7		*Salmonella* Typhimurium		*Staphylococcus aureus*		*Listeria monocytogenes*	
Treatment	High	Low	High	Low	High	Low	High	Low
Before treatment	5.52 ± 0.35a	3.85 ± 0.07a	5.48 ± 0.26a	3.97 ± 0.10a	5.86 ± 0.34a	3.82 ± 0.02a	5.81 ± 0.46a	3.64 ± 0.09a
Tap water	5.36 ± 0.32a (0.16)	3.45 ± 0.02b (0.40)	4.96 ± 0.37a (0.52)	3.58 ± 0.07b (0.39)	5.64 ± 0.38ab (0.22)	3.47 ± 0.03b (0.35)	5.66 ± 0.45ab (0.15)	3.51 ± 0.03a (0.13)
NaOCl	5.09 ± 0.17a (0.43)	2.91 ± 0.09 cd (0.94)	4.25 ± 0.48b (1.23)	2.84 ± 0.11de (1.13)	4.80 ± 0.07c (1.06)	3.08 ± 0.03d (0.74)	4.45 ± 0.34d (1.36)	2.94 ± 0.09cd (0.70)
NEW	4.53 ± 0.36b (0.99)	2.51 ± 0.17e (1.34)	3.82 ± 0.20b (1.66)	2.84 ± 0.06de (1.13)	5.09 ± 0.54c (0.77)	2.79 ± 0.03f (1.03)	5.30 ± 0.49bc (0.51)	2.95 ± 0.08cd (0.69)
AlEW	4.52 ± 0.32b (1.00)	2.81 ± 0.15cd (1.04)	3.72 ± 0.20b (1.76)	3.09 ± 0.04c (0.88)	5.40 ± 0.65bc (0.46)	2.91 ± 0.03e (0.91)	5.33 ± 0.41bc (0.48)	3.05 ± 0.12bc (0.59)
AcEW	4.47 ± 0.32b (1.05)	2.48 ± 0.08e (1.37)	3.58 ± 0.20b (1.90)	2.56 ± 0.04f (1.41)	4.99 ± 0.62c (0.87)	2.73 ± 0.03g (1.09)	5.04 ± 0.43cd (0.77)	2.85 ± 0.13d (0.79)
PAA	4.56 ± 0.09b (0.96)	2.71 ± 0.19de (1.14)	4.01 ± 0.34b (1.47)	2.79 ± 0.23ef (1.18)	4.41 ± 0.02c (1.45)	2.81 ± 0.03f (1.01)	4.50 ± 0.09d (1.31)	2.67 ± 0.12e (0.97)
HP	5.34 ± 0.08a (0.18)	3.01 ± 0.12c (0.84)	4.76 ± 0.21a (0.72)	3.04 ± 0.08cd (0.93)	5.75 ± 0.20a (0.11)	3.32 ± 0.03c (0.50)	5.48 ± 0.12b (0.33)	3.13 ± 0.02b (0.51)

Each value is the mean of three replicates with the standard deviation in three independent experiments. Any two means in the same row followed by the same letter for each strain are not significantly (*P* > 0.01) different by Tukey's multiple range tests. The log difference in the viable cell counts after treatment compared to the initial inoculation is expressed as the number in the parenthesis. NaOCl, 100 ppm sodium hypochlorite (standard industrial practice); PAA, 50 ppm peracetic acid; HP, 1% hydrogen peroxide; AcEW, acidified electrolyzed water (pH 2.71 ± 0.19, 1,152 ± 20 ORP, 111.6 ± 15.7 ppm of free chlorine); NEW, neutral electrolyzed water (pH 8.43 ± 0.02, 670 ± 23 ORP, 99.6 ± 7.4 ppm of free chlorine); AlEW, alkaline electrolyzed water (pH 10.43 ± 0.42, 211 ± 13 ORP, 52.9 ± 13.8 ppm of free chlorine); high, the initial inoculation level of the bacteria was ∼10^5^–10^6^ CFU g^−1^; low, the initial inoculation level was ∼10^3^–10^4^ CFU g^−1^.

A different initial inoculation size did not affect the efficacy of several sanitizers, such as sodium hypochlorite, AcEW, NEW, and AlEW, which was similar to other study (Abadias et al. 2008b). After the above pretreatments, the inoculated bacteria were reduced by ∼1 log CFU g^−1^, regardless of the initial microbial level. This result indirectly indicates that bacteria may penetrate into internal spaces of vegetable leaves during contamination. After bacterial penetration inside a leaf has occurred, sanitizers are unable to access and inactivate the bacteria, even though a low population of bacteria exists on fresh produce (Abadias et al. 2008b; Lynch et al. 2009). Among the bacteria tested, the reduction in *S*. Typhimurium was higher at the high initial load than on the low initial load, with a 0.3–0.9 log CFU g^−1^ difference. For *E. coli* O157:H7, however, the reduction achieved was slightly greater at the low initial dose. Relatively high reduction in *S*. Typhimurium at high initial load was presumably attributed to the microbial characteristics that have a similar preference for the intact surface and cut edges and to the greater opportunity for contact between the microbes and the sanitizer (Takeuchi et al. 2000; Koseki et al. 2003). The present results can provide some useful information for the fresh-cut industry to select an appropriate disinfectant such as AcEW, 50 ppm peroxyacetic acid, and NEW to efficiently reduce the harmful microorganisms associated with fresh-cut vegetables and to still keep good sensory quality after treatment.
